# One problem, too many solutions: How costly is honest signalling of need?

**DOI:** 10.1371/journal.pone.0208443

**Published:** 2019-01-11

**Authors:** Szabolcs Számadó, Dániel Czégel, István Zachar

**Affiliations:** 1 CSS-RECENS, MTA Centre for Social Science, Tóth Kálmán u. 4., Budapest, Hungary; 2 Evolutionary Systems Research Group, MTA, Centre for Ecological Research, Hungarian Academy of Sciences, Tihany, Hungary; 3 Department of Plant Systematics, Ecology and Theoretical Biology, Eötvös Loránd University, Budapest, Hungary; 4 Deptartment of Brain and Cognitive Sciences, Massachusetts Institute of Technology, Cambridge, MA, United States of America; 5 Center for the Conceptual Foundations of Science, Parmenides Foundation, Kirchplatz 1, Pullach, Munich, Germany; 6 MTA-ELTE Theoretical Biology and Evolutionary Ecology Research Group, Eötvös Loránd University, Department of Plant Taxonomy and Ecology, Budapest, Hungary; University of Notre Dame, UNITED STATES

## Abstract

The “cost of begging” is a prominent prediction of costly signalling theory, suggesting that offspring begging has to be costly in order to be honest. Seminal signalling models predict that there is a unique equilibrium cost function for the offspring that results in honest signalling and this cost function must be proportional to parent’s fitness loss. This prediction is only valid if signal cost and offspring condition is assumed to be independent. Here we generalize these models by allowing signal cost to depend on offspring condition. We demonstrate in the generalized model that any signal cost proportional to the fitness gain of the offspring also results in honest signalling. Moreover, we show that any linear combination of the two cost functions (one proportional to parent’s fitness loss, as in previous models, the other to offspring’s fitness gain) also leads to honest signalling in equilibrium, yielding infinitely many solutions. Furthermore, we demonstrate that there exist linear combinations such that the equilibrium cost of signals is negative and the signal is honest. Our results show that costly signalling theory cannot predict a unique equilibrium cost in signalling games of parent-offspring conflicts if signal cost depends on offspring condition. It follows, contrary to previous claims, that the existence of parent-offspring conflict does not imply costly equilibrium signals. As an important consequence, it is meaningless to measure the “cost of begging” as long as the dependence of signal cost on offspring condition is unknown. Any measured equilibrium cost in case of condition-dependent signal cost has to be compared both to the parent’s fitness loss and to the offspring’s fitness gain in order to provide meaningful interpretation.

## Background

Parent-offspring communication is a hotly debated topic appearing continuously in the forefront of behavioural sciences [1–4]. On the one hand, there is a conflict of interests between the involved parties. Yet, despite this obvious conflict [5], offspring frequently solicit food from the parents. In general, this solicitation is found to be honest as more needy offspring beg more intensively [6]. Game theoretical explanations of begging behaviour gained much attention over the years [7–16]. Most of these game theoretical models predicted costly signalling [7], which became the dominant expectation in past decades.

Nöldeke and Samuelson [17] offered an enlightening account of the cost of honest signalling of need, based on Godfray’s model [7]. They have demonstrated that at equilibrium (where honest signalling exists), the signalling cost of the offspring is proportional to the fitness loss of the parent resulting from the transfer of resources. They also showed that the factor of proportionality is solely determined by the degree of relatedness between parent and offspring. Consequently, they claimed that the offspring's condition (and its expected benefit due to the received resource) influences the signalling cost only to the extent that it influences the parent's loss of fitness. A key assumption of their model (and of the original Godfray model [7]) is that signal cost and offspring condition assumed to be independent: “*We concentrate on the signaling of need rather than quality*, *meaning that the sender's condition does not affect the cost of signaling in our model but does affect the benefits conferred by the receiver's actions*” ([17] p. 527). However, this assumption is unnecessarily restricting and is lacking strong empirical support. Here we provide a more general version of these models by allowing for signal cost to depend on offspring condition. We prove that under this assumption there exists another equilibrium with honest signalling, which can be readily derived from their equations [17]. At this second equilibrium, the cost of signalling is proportional to the expected fitness benefit of the offspring, and (analogously to the other case) the parent's fitness loss affects the signalling cost only to the extent it affects the offspring’s gain. Moreover, we demonstrate that any linear combination of these two cost functions provides an equilibrium with honest signalling. Thus, there is an infinite number of distinct equilibria (in terms of offspring’s signal cost) where honest signalling exists.

## Methods

Nöldeke and Samuelson [17] have designed their model based on the seminal work of Godfray [7]. They have calculated the fitness functions of the two parties, parent and offspring. The parent is interested in the condition of the offspring to transfer the least amount of resource to maximize its own inclusive fitness (all future offspring included) whereas the offspring is interested in receiving the largest possible amount of resource to maximize its own inclusive fitness (all future siblings included). The offspring’s condition is described by a positive continuous variable (*c*). The requirement for signalling stems from the fact that the parent cannot assess this condition directly. The offspring, however, can opt to engage in communication with a potentially costly signal (*x*). In the original model of Nöldeke and Samuelson, *x* denoted both the *level* (intensity) and the *cost* of the signal [17]. Here, we distinguish between these two by denoting the intensity of the signal by *x* and allowing the cost of signal *f*(*x*) to depend on the intensity in an arbitrary way. This allows a more general interpretation of the model and it avoids potential technical pitfalls.

The parent has control over *Z* amount of resource that the parent must divide between the offspring and itself, where the offspring receives part *z* of *Z* and the parent retains part *y* = *Z – z*. The inclusive fitness functions of offspring and parent (*v* and *u*, respectively, after [17]) are:

v(c,x,z)=h(c,z)−f(x)+ψg(Z−z), (Eq 1)

u(c,x,z)=γ(h(c,z)−f(x))+g(Z−z), (Eq 2)

where *h*(*c*, *z*) and *g*(*Z* – *z*) are the direct fitness gains of offspring and parent, respectively, when *z* amount of resource is transferred to offspring. Both *h* and *g* are assumed to be differentiable and increasing functions (accordingly strictly decreasing with *z*). The coefficient of relatedness between current offspring (and any future siblings from the parent) is denoted by *ψ*; the coefficient of relatedness of the parent to its current (and future) offspring is denoted by *γ*. The offspring strategy is the level of solicitation *x* as function of the offspring’s condition *c*, whereas the parental strategy is the amount of resource shared *z* as a function of offspring solicitation *x*.

### Conditions of the honest signalling equilibrium

A stable equilibrium of honest signalling requires three conditions to be met: (*i*) signals must be honest, (*ii*) parents have to respond to signals and (*iii*) the equilibrium must be evolutionarily stable. The latter condition implies that there is a pair of optimal parent and offspring strategies (z*(*x*), x*(*c*)) from which it does not worth departing unilaterally for any of the participants [17]. At an honest equilibrium, parents know the condition of the offspring as their signal of need directly corresponds to offspring’s level of need. Thus, the parent’s equilibrium strategy has to maximize the parent’s inclusive fitness *u* for any given *c*, i.e. the following inequality must hold:

$$u(c,x^{*}(c),z^{*}(x^{*}(c)))\geq u(c,x^{*}(c),z(x^{*}(c))),$$ (Eq 3)

where x* is the equilibrium signal by the offspring, depending on its own condition and z* is the parent’s equilibrium transfer depending on offspring’s signal intensity. Substituting Eq 2 Into Eq 3 gives the following condition:

$$\gamma (h(c,z^{*}(x^{*}(c)))-f(x^{*}(c)))+g(Z-z^{*}(x^{*}(c)))\geq \gamma (h(c,z(x^{*}(c)))-f(x^{*}(c)))+g(Z-z(x^{*}(c))).$$ (Eq 4)

Analogously to parent, offspring’s equilibrium strategy is to maximize its own inclusive fitness *v* given the parental equilibrium strategy z*(*x*) and the condition of the offspring *c*. Thus, the following condition must hold for any *c* and *x* (Eq 2 at [17]):

$$v(c,x^{*}(c),z^{*}(x^{*}(c)))\geq v(c,x,z^{*}(x)).$$ (Eq 5)

Substituting into Eq 1 gives the following condition:

$$h(c,z^{*}(x^{*}(c)))-f(x^{*}(c))+\psi g(Z-z^{*}(x^{*}(c)))\geq h(c,z^{*}(x(c)))-f(x(c))+\psi g(Z-z^{*}(x(c))).$$ (Eq 6)

In a signalling equilibrium, the parent’s transfer for all *c* must satisfy (Eq 5 at [17]):

$$z^{*}(x^{*}(c))=\tilde{z}(c),$$ (Eq 7)

where x* denotes the offspring’s equilibrium signal intensity, z* the parent’s equilibrium transfer function, and $\tilde{z}(c)$ the parent’s optimal transfer function.

## Results

The argument of Nöldeke and Samuelson [17] is as follows: the cost of signal at equilibrium has to dispense the conflict of interest between parent and offspring. Accordingly, the two solution functions of *h* and *g* of the optimization problems of parent and offspring have to give the same result (see [18] for more general results). In the absence of signalling cost, at the maximum of the offspring’s inclusive fitness, the following conditions must be met:

$$h_{z}(c,z)-\psi g_{y}(Z-z)=0,$$ (Eq 8)

$$h_{z}(c,z)-\frac{1}{\gamma }g_{y}(Z-z)=0,$$ (Eq 9)

where subscripts denote derivatives with respect to the subscripted variable. At the optimum, the derivatives of the two components of the fitness gain must equal (Eqs 7 and 8 at [17]):

$$h_{z}(c,z)=\psi g_{y}(Z-z),$$ (Eq 10)

$$h_{z}(c,z)=\frac{1}{\gamma }g_{y}(Z-z).$$ (Eq 11)

**Fig 1:**
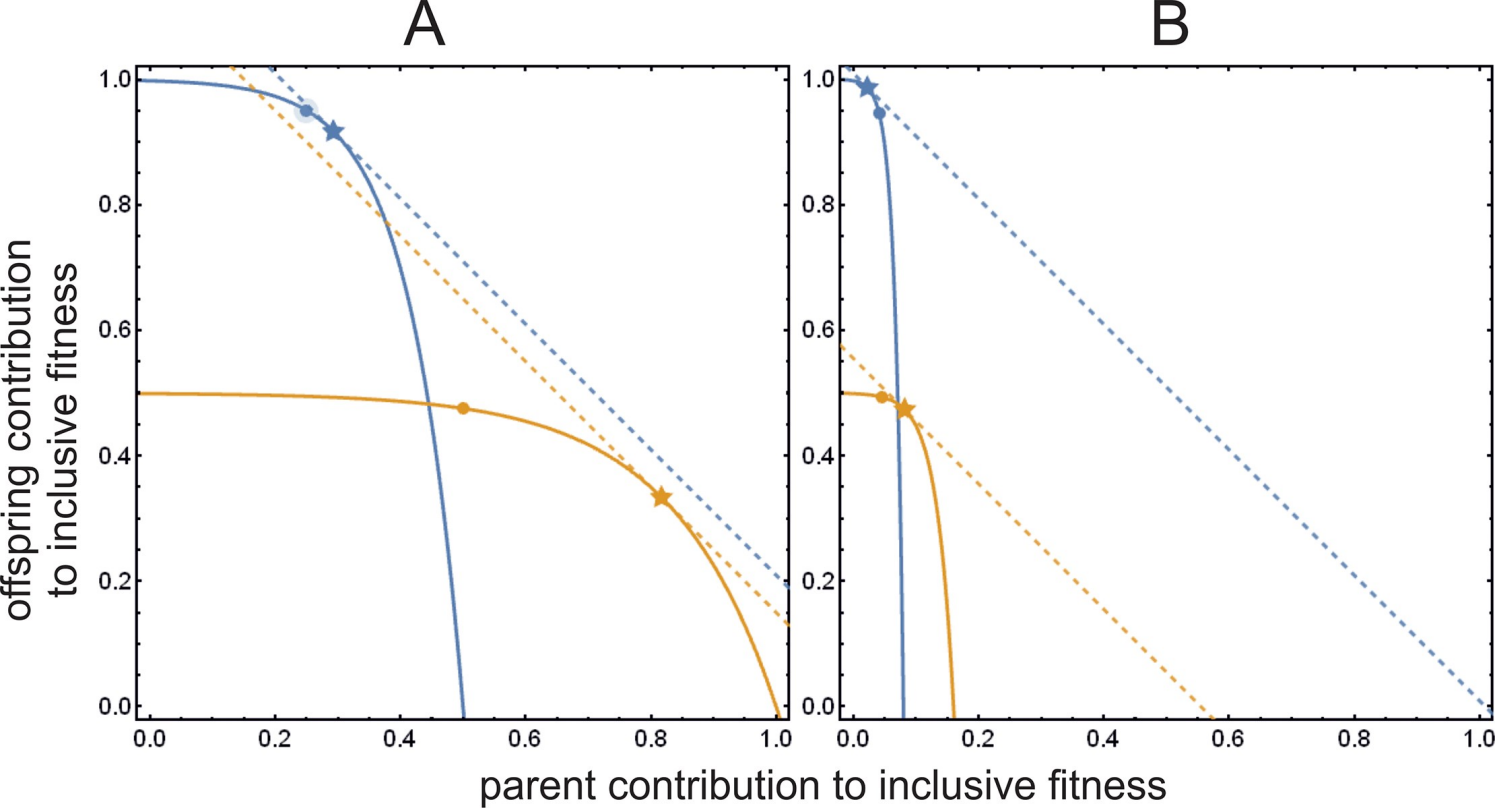
Inclusive fitness functions and optima without signalling cost. (A) *G* = ½; (B) *G* = 0.08 (as in [7]). Inclusive fitness functions parameterized by *z* are shown as yellow curve for parent’s (function *u* according to Eq 2 without signal cost *f*(*x*)) and (blue curve for offspring (function *v* according to Eq 1 without signal cost *f*(*x*)). The *x* coordinate value of parent’s curve is the parent’s own fitness contribution *g*(*c*, *z*), the *y* coordinate value is the fitness contribution the offspring (*γ h*(*c*, *z*)); similarly, the *x* value of the offspring’s curve is the parent’s contribution (*ψ g*(*z*)), the *y* value is the offspring’s own fitness *h*(*c*, *z*). The actual inclusive fitness value is the sum of the appropriate coordinate values, both for parent and offspring. Parameters are *Z* = 2, *γ* = 1/2, *ψ* = ½, *U* = 1, *c* = 3. Yellow and blue stars indicate parent’s and offspring’s optima, respectively. Dashed lines are the calculated derivative tangents that touch optima at 45°, indicating maximum fitness. The optimum *z* value for parent and offspring are not identical: the yellow dot indicates what the parent’s fitness is at the offspring’s optimum *z*; blue dot is the offspring’s fitness in case of parent’s optimum *z*.

Clearly, the marginal fitness gain of the offspring (in the absence of signal cost) is different from the offspring’s point of view (Eq 10) than from the parent’s point of view (Eq 11), hence parent and offspring maximize different functions. Thus, there is a clear conflict of interest between them. An illustration of this conflict and the corresponding trade-offs are illustrated by Fig 1. The shape of these trade-offs is different since the weights of the parental fitness component (*g*) and the offspring fitness component (*h*) are different for the offspring and the parent. The fitness components of the inclusive fitness of the offspring and the parents change alongside the blue and yellow curves, respectively, with increasing *z*. The trade-off implies that one component cannot be increased without the loss of fitness in the other component. Blue and yellow stars represent optimal resource allocation and blue and yellow dots indicate the position (fitness) of the offspring and the parent, respectively, when the resource allocation is optimal for the other party. Clearly the dots do not overlap with the stars, hence the optimal resource allocation of one party is not optimal for the other.

Nöldeke and Samuelson [17] proposed that the cost of signals should resolve this conflict in the honest signalling equilibrium. They proposed the specific cost function which we denote by *L*_1_(*z*) here:

$$L_{1}(z)=g(Z-z^{0})-g(Z-z),$$ (Eq 12)

where *z*^0^ is the resource requirement of the offspring in the least needy condition, that is $z^{0}=\min_{c}\;\tilde{z}(c)$ [17]. The cost at equilibrium is:

$$f_{1}(x^{*}(c))=\left(\frac{1}{\gamma }-\psi \right)(g(Z-z^{0})-g(Z-\tilde{z}(c)))=\left(\frac{1}{\gamma }-\psi \right)L_{1}(\tilde{z}(c)),$$ (Eq 13)

where $\frac{1}{\gamma }-\psi$ defines the magnitude of the parent-offspring conflict. In equilibrium, $z=\tilde{z}(c)$. The relationship between *f*(*x*) and *L*(∙) discussed further in Section A in S1 Text.

So far, we have followed the design of Nöldeke and Samuelson [17]. However, starting from the same equations (Eqs 8 and 9), a different cost function of signalling can also be obtained. Instead of providing the optimality conditions to calculate the offspring’s marginal fitness gain, one can rearrange Eqs 8 and 9 differently to calculate the parental marginal fitness gain, from the offspring’s point of view (without signal cost):

$$g_{z}(Z-z)=\frac{1}{\psi }h_{z}(c,z),$$ (Eq 14)

and from the parent’s point of view:

$$g_{z}(Z-z)=\gamma h_{z}(c,z).$$ (Eq 15)

Clearly, in the absence of signal cost, the marginal fitness gain of the parent (as a function of resource allocation) is different from the offspring’s point of view (Eq 14) than from the parent’s point of view (Eq 15). This still implies the conflict of interest. Following the same logic as above, at the honest signalling equilibrium, these equations have to provide the same results. That is, the parent’s optimum has to be the same, viewed either from the offspring’s or from the parent’s aspect. Thus, just as before, the difference between the right-hand sides of Eqs 14 and 15 gives the cost that has to be subtracted from the offspring fitness so that the two equations result in the same optimum. The cost function we propose is:

$$L_{2}(c,z)=h(c,z)-h(c,z^{0}),$$ (Eq 16)

and the cost at equilibrium is:

$$f_{2}(x^{*}(c))=(1-\gamma \psi )(h(c,\tilde{z}(c))-h(c,z^{0}))=(1-\gamma \psi )L_{2}(c,\tilde{z}(c)).$$ (Eq 17)

The existence of the signalling equilibrium can be proved as before (see Section B in S1 Text).

So far, we have proved that there are two honest signalling equilibria corresponding to two different cost functions. Since each of these cost functions can remove the conflict of interest between parent and offspring, it follows that any linear combination of these functions is also a solution to the optimization problem. Thus, the general cost function of the optimum strategies is as follows:

$$L(c,z)=\alpha \left(\frac{1}{\gamma }-\psi \right)L_{1}(z)+(1-\alpha )(1-\gamma \psi )L_{2}(c,z).$$ (Eq 18)

The cost at equilibrium is:

$$f(x^{*}(c))=\alpha \left(\frac{1}{\gamma }-\psi \right)L_{1}(\tilde{z}(c))+(1-\alpha )(1-\gamma \psi )L_{2}(c,\tilde{z}(c)).$$ (Eq 19)

**Fig 2:**
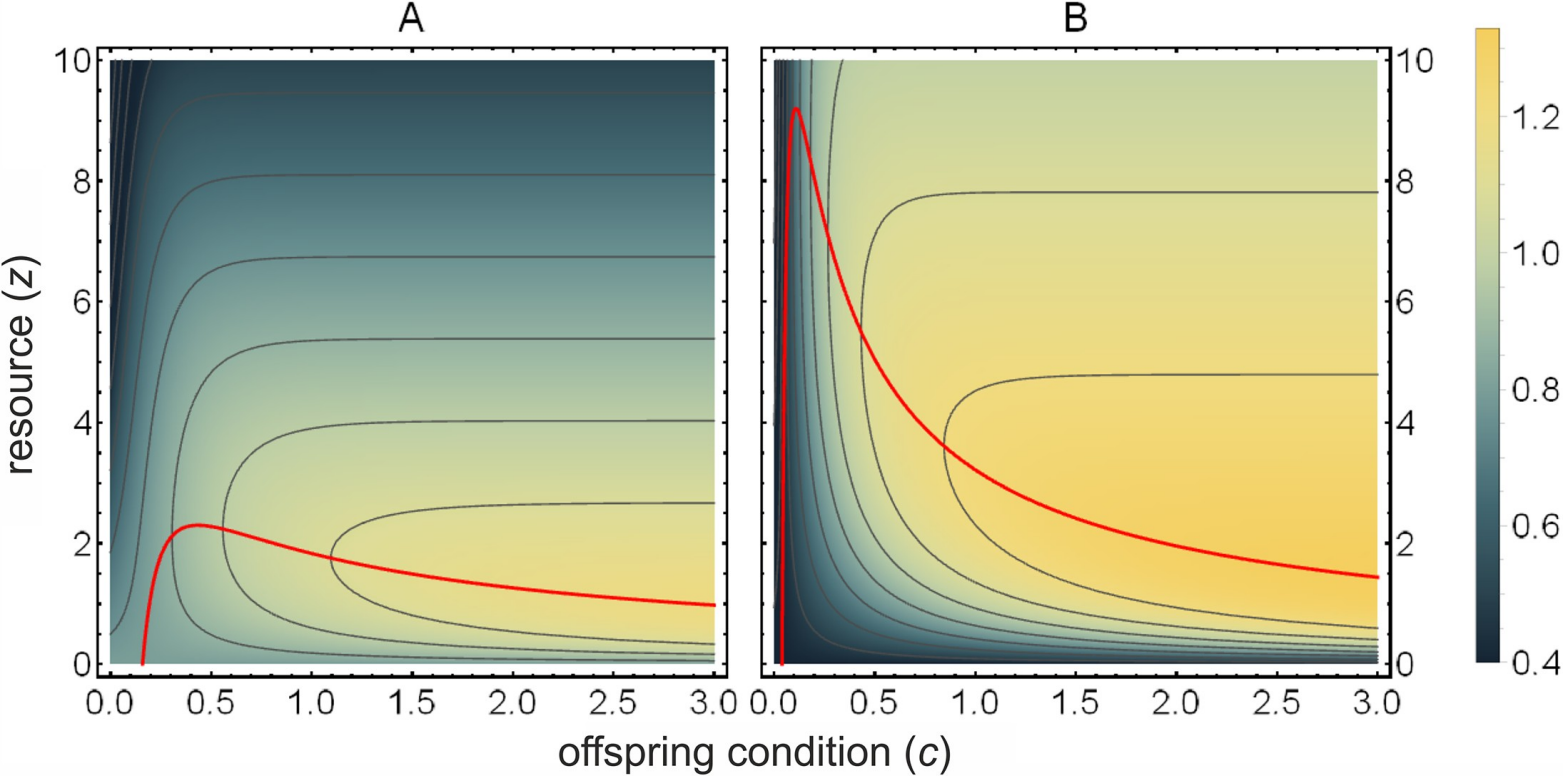
Inclusive fitness depending on offspring condition *c* and parental investment *z*. (A) Parental fitness (function *u* according to Eq 2 without signal cost *f*(*x*)). (B) Offspring fitness (function *v* according to Eq 1 without signal cost *f*(*x*)). Red line connects the equilibrium *z* values where $\tilde{z}=\ln\left(\frac{\gamma Uc}{G}\right)/c$ holds. Parameters are *Z* = 10, *G* = 0.08, *U* = 1, *γ* = ½, *ψ* = ½.

Finally, we provide a numerical example using Godfray's [7] equations. He used the following equations for the offspring’s and parent’s fitness contributions, respectively:

h(c,z)=U(1−exp(−cz)), (Eq 20)

g(Z−z)=G(Z−z), (Eq 21)

where *U* and *G* are constants. From now on, we use the values provided by Godfray [7]: *U* = 1, *G* = 0.08. Fig 1 shows the actual inclusive fitness values for offspring and parent (*v* of Eq 1 and *u* of Eq 2, respectively) when there is no cost of signalling, as functions of the condition of the offspring *c* and parental resource allocation *z*. Fig 2 also shows the equilibrium transfer function for parent and for offspring (red curve) without signal cost, which corresponds to the optimal resource allocation for the offspring and the parent, respectively (as a function of *c*). Fig 2 clearly demonstrates that the optima are at different *z* values for the two parties.

Substituting Godfray’s equation (Eqs 20 and 21) into the cost function defined by *L*_*1*_ (Eq 12) yields:

$$L_{1}(z)=\left(\frac{1}{\gamma }-\psi \right)G(z-z^{0}).$$ (Eq 22)

Substituting the same equations into the cost function defined by *L*_2_ (Eq 16), yields:

$$L_{2}(c,z)=(1-\gamma \psi )U(\exp\left(-cz^{0}\right)-\exp\left(-cz\right)).$$ (Eq 23)

**Fig 3:**
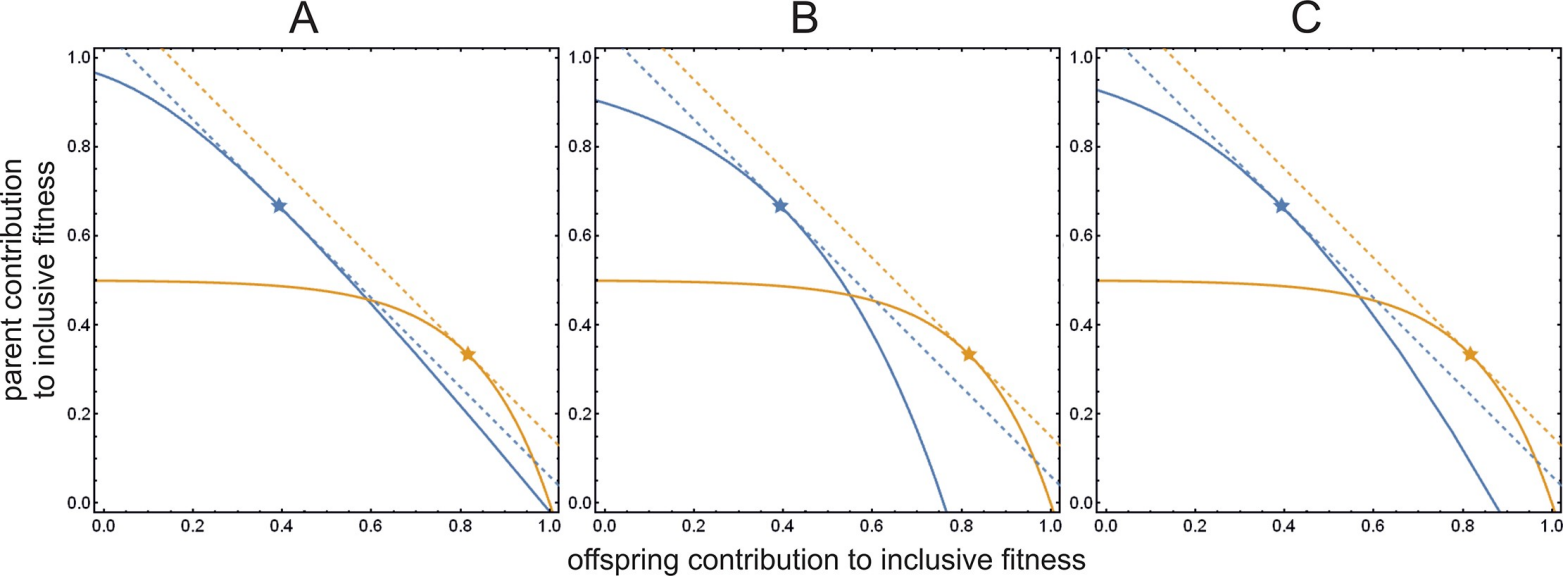
Inclusive fitness functions and optima with signalling cost. Inclusive fitness functions, parameterized by *z*, are represented by the yellow curve (for the parent, fitness function *u* according to Eq 2) and by the blue curve (for the offspring, fitness function *v* according to Eq 1). The *x* coordinate value of parent’s curve is the parent’s own fitness contribution *g*(*c*, *z*), the *y* coordinate value is the fitness contribution of all future offspring (*γ h*(*c*, *z*)); similarly, the *x* value of the offspring’s curve is the parent’s contribution (minus cost) (*ψ g*(*z*) – *L*(*α*, *c*, *z*)), the *y* value is the offspring’s own fitness *h*(*c*, *z*). The actual inclusive fitness value is the sum of the appropriate coordinate values, both for parent and offspring. Parameters are *Z* = 2, *γ* = ½, *ψ* = ½, *U* = 1, *G* = ½, *c* = 3. Yellow and blue stars indicate parent’s and offspring’s fitness optima. Dashed lines are the calculated derivative tangents that touch optima at 45°, indicating maximum fitness. The optimum *z* value for parent and offspring are always identical, regardless of *α* and *β* values. (A) Cost function *L*_1_ of Nöldeke and Samuelson [17] (Eq 12; *α* = 1). (B) Cost function *L*_2_ introduced in this paper (Eq 16; *α* = 0). (C) Linear combination of the above two cost functions *L*_1_ and *L*_2_ (*α* = ½) (Eq 18).

Fig 3 shows the same trade-off as Fig 1 but with the cost function included in the offspring inclusive fitness (Eqs 1 and 2). Fig 3A shows the new cost function, Fig 3C shows the cost function proposed by Nöldeke and Samuelson [17], and Fig 3B shows a linear combination of the two functions (with a weight of *α* = 0.5). The amount of transferred resource at the equilibrium corresponds both with parent and offspring optima (dots overlap with stars). This effectively means that these cost functions indeed remove the conflict of interest present between parent and offspring. In the Supplementary Material we provide interactive version of these figures (as a Mathematica notebook in S1 Notebook) that can be used to interactively explore parameter ranges with or without signal cost and with various linear combinations of the two cost functions *L*_1_ and *L*_2_. We also provide a movie that summarizes the interactive document (S1 Video).

**Fig 4:**
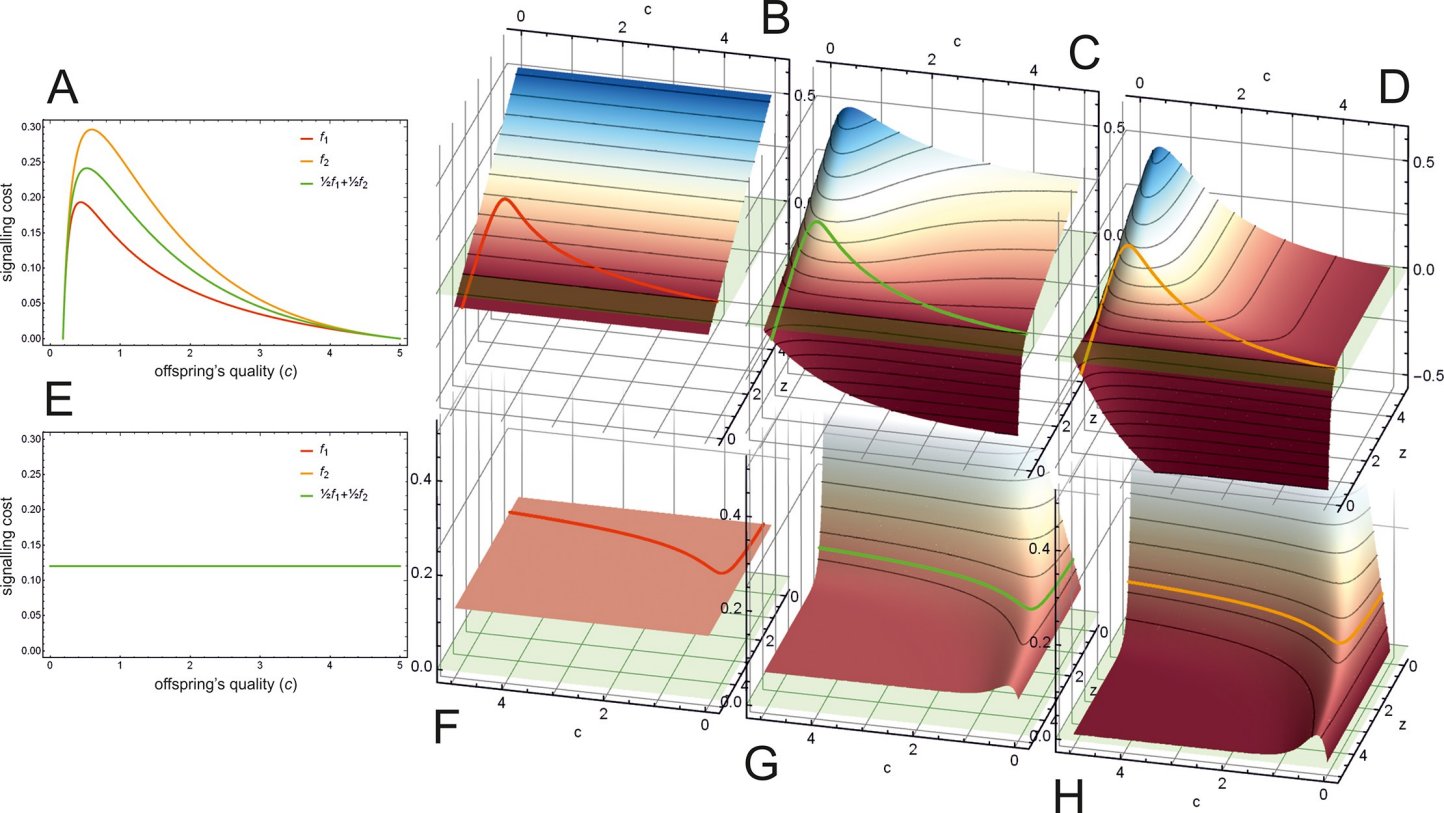
Signalling cost functions, depending on offspring condition *c* and parental transfer *z*. (A) equilibrium signal cost (*f*(x*(*c*))) for the different cost functions (Eqs 13, 17 and 19). (B) Signal cost function *L*_1_ (Eq 12). (C) Linear combination ½ *L*_1_ + ½ *L*_2_ (Eq 18) (D) signal cost function *L*_2_ introduced in this paper (Eq 16). Red, green and orange curves show the signal cost (*f*(x*(*c*))) along the equilibrium path (which describes the equilibrium transfer function $\tilde{z}(c)$ for parent as a function of *c*); panel **A** shows these curves projected to the *c*-*f*(c) plane. (E) Partial derivatives of the signal cost functions *L*_1_, *L*_2_ and their linear combination, with respect to *z* along the equilibrium path as a function of *c*. (F) Partial derivative of signal cost function *L*_1_ (Eq 12) with respect to *z*. (G) Partial derivative of the linear combination ½ *L*_1_ + ½ *L*_2_ (Eq 18) (H) Partial derivative of signal cost function *L*_2_ introduced in this paper (Eq 16) with respect to *z*. Red, green and orange curves show the partial derivatives of the respective signal cost functions along the equilibrium path with respect to *z*; panel **E** shows these curves projected to the *c*-*f*(*c*) plane. Parameters are *Z* = 10, *G* = 0.08, *U* = 1, *γ* = ½, *ψ* = ½.

Fig 4 shows the actual values for the different cost functions *L*_1_ (Fig 4B), *L*_2_ (Fig 4D) and their linear combination (Fig 4C), when using Godfray’s equation (Eqs 20 and 21). Red, yellow and green curves show the signal cost along the equilibrium path (*f*_1_(x*(*c*)) and *f*_2_(x*(*c*))) (Eqs 13 and 17). This equilibrium cost can be calculated by substituting *z* with the amount of optimal parental investment $\tilde{z}=\ln\left(\frac{\gamma Uc}{G}\right)/c$ into Eqs 22 and 23. Fig 4A shows how these equilibrium cost functions compare to each other as functions of offspring condition *c*. Note, that while the absolute value of the equilibrium signal cost is different for each cost function, the partial derivative with respect to *z* is the same along the equilibrium path (see Fig 4F, 4G and 4H). Fig 4E illustrates this effect.

**Fig 5:**
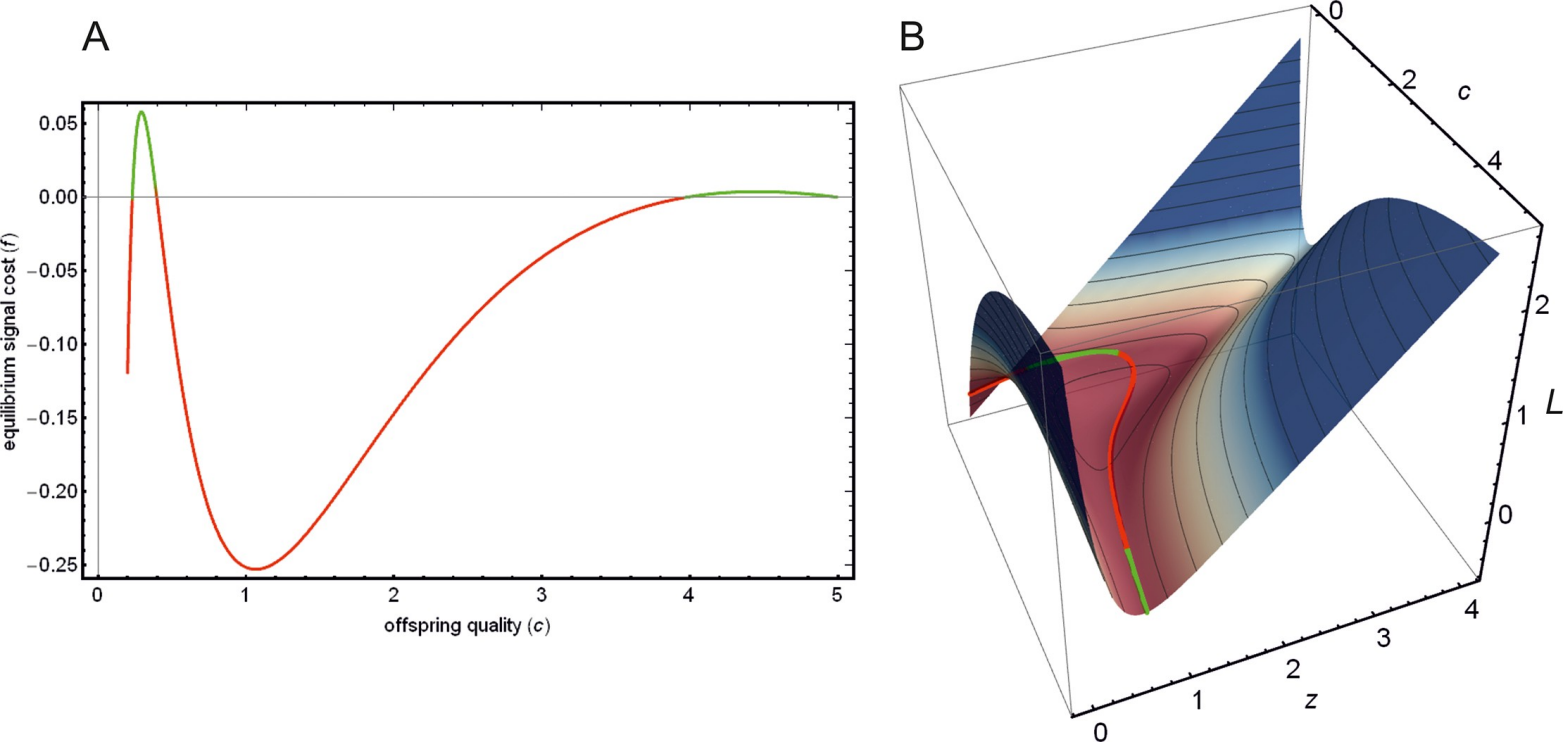
Negative equilibrium cost. (B) Cost of signalling *L* depending on offspring condition *c* and amount of transferred resource *z* (Eq 18). Red-green curve along the surface (and in inset) indicates equilibrium cost function (f(x*(*c*))) at equilibrium $\tilde{z}$ (Eq 19), where red sections indicate negative cost values. (A) shows this curve projected to the *c*-*f*(c) plane. Parameters are: $\left\{Z=4,G=0.1,U=1,\gamma =\frac{1}{2},\psi =\frac{1}{2},c_{max}=5,\alpha =5\right\}$.

Finally, Fig 5 shows a cost function where the equilibrium cost is negative for some of the signallers. Such cost functions can be generated when the value of *α* is greater than one (Eq 18; e.g. *α* = 5 in this case).

## Discussion

According to Nöldeke and Samuelson [17] (and Eq 15), based on Godfray’s original differential benefit model [7], the cost of honest signalling should be proportional to the parent’s fitness loss. A key assumption of their model is that the cost of signalling is independent of offspring condition, and the key insight is that parents can still enforce honest signalling outcome by fine tuning their provisioning rule. This is a simple and elegant idea, however, one might wonder if it is the only solution that yields honest signalling in equilibrium. While the existence of an infinite number of costly equilibria is known in general [18, 19], no other equilibrium has been calculated yet in terms of Godfray’s model. Here we have shown that under the assumption of condition dependent signal cost, a second cost function exists when the cost is proportional to offspring's fitness gain, which also yields a signalling equilibrium with costly signals. Furthermore, we have demonstrated, that any linear combination of the two extremal cost functions is an equilibrium itself, which effectively proves that an infinite number of honest, evolutionarily stable costly signalling equilibria exist for Godfray’s model. Moreover, it is possible to show that there are linear combinations of cost functions where the equilibrium cost is actually negative for some of the signallers. While we have specifically derived the second extremum cost function for Godfray’s model, our results have important theoretical and empirical implications that apply to the general case of the signalling of need, as discussed below.

There are three important conclusions of past research (*i*-*iii*) and two major outcomes of our results (*iv*-*v*), concerning the signalling of need which apply generally: (*i*) honest signalling need not be evolutionarily stable [16]; (*ii*) there is, on average, a shared interest between parent and offspring, hence partially honest pooling equilibria can exist with cost-free signals [11, 13]; (*iii*) there exists an honest signalling equilibrium in a differential benefit model [7], where the cost of signalling is proportional to the parent's fitness loss [17]. As we have shown in this paper, (*iv*) there exists an additional honest signalling equilibrium allowing signal cost to depend on offspring condition (i.e. differential cost model), in which the cost of signalling is proportional to the offspring's expected fitness gain; (*v*) there is an infinite number of honest signalling equilibria where the cost of signalling is proportional to the linear combination of the cost functions of (*iii*) and (*iv*), including equilibria where the cost of signalling is smaller–even negative for some signallers–than in any other equilibria. All in all, both differential benefit and differential cost models can explain honest signalling, yet they have divergent predictions. It is possible, that a differential cost model offers a better fit for the empirically observed patterns of parent-offspring communication than differential benefit models (marginally mentioned in [20]). This could open up possibilities for other cost-free [19, 21, 22] or even negative-cost equilibria [22].

There is another important implication of our results and the above considerations: it is not possible to decide in case of a real populations based on game theoretical models alone in which one of the infinite numbers of costly honest equilibria has the population been settled to, or which one it can reach (provided that an honest separating equilibrium exists). In order to answer questions of which evolutionary trajectory will be (or have been) played out, a more dynamic approach is needed. Godfray and Johnstone [10] has calculated the fitness advantage of the signalling equilibrium over the non-signalling equilibrium using the cost function of Nöldeke and Samuelson [17]. Our results could significantly change the outcome of these calculations, profoundly affecting the evolutionary consequences. This is left for future work.

Since the publication of Godfray’s [7] influential model, a lot of empirical research has been carried out to measure the “cost of begging”. It was realized very early that the metabolic cost of begging is not unreasonably high [23–25] therefore it probably does not fit the predictions of costly signalling theory. Attempts to try to measure the cost of increased begging afflicted on growth provided mixed results [26–28]. However, several types of other costs were proposed, e.g. predation risk [29–31], immunological [32–34] or oxidative costs [35]; for a review, see [36]. We must emphasize, that measuring any cost *in absolute value* is not sufficient [19, 37]: any measured cost has to be *compared* to something, i.e. only relative measures are informative. One of the reasons why current empirical results are inconclusive is that we don’t have any information about how these costs relate to the benefits of the parties, though see Moreno-Rueda and Redondo [34] for an exception.

The most important conclusion of the current investigation is that a condition-dependent signal cost allows other equilibria than one independent of condition. This begs the question: which type of model is more relevant? Are begging signals condition-dependent or independent in real populations? Clearly, whichever appears more often in nature should be the relevant assumption in a model. Unfortunately, there is little information on the condition-dependence of begging signals as most experimental studies do not have an experimental treatment based on condition. Some studies, however, provide indirect evidence that begging cost could be condition-dependent. For example, tadpoles of the frog *Oophaga pumilio* beg differently in the absence of a predator when hungry or satiated (hungrier tadpoles beg more, [38]). In another study, yellow-legged gull chicks (*Larus michahellis*) gave more chatter calls per time when supplemented with vitamin E (antioxidant) than the control treatment [39], which suggests that the cost of giving these calls might have been lowered in the vitamin E-supplemented group (i.e. good condition). However, in great tits (*Parus major*), supplementing vitamin E had no effect on begging intensity [40, 41]. Finally, nestlings of carotinoid-supplemented great tit parents begged more intensely [42]. As one can see, studies based on condition-dependence are scarce and inconclusive. The ongoing omission of condition-dependence is at least puzzling in a field that set out to study signals of need. We suspect that this is due to the influence of seminal papers that assumed condition-independent signal costs early on and consequently influenced empirical research in that direction. While one can only applaud the simplicity and elegance of the original idea, the same simplicity might have masked a fundamental question that needs to be resolved.

The results of Nöldeke and Samuelson [17] and the results presented here, along with other theoretical results [19, 22], provide a guide on how one can meaningfully compare the costs of the different parties involved. It follows that (field) researchers, when testing the predictions of costly begging models, have to take into account (i.e. measure) both the potential fitness loss of the parent *and* the potential fitness gain of the offspring.

We have demonstrated that Godfray’s famous “cost of begging” prediction holds only in signalling systems where signal cost is independent of condition. Consequently, it is incorrect (and possibly misleading) to unconditionally generalize this prediction to signalling systems of condition-dependent costs. The first step of any empirical investigation should be to establish the condition dependence of signal cost as equilibria depend on this property. Unfortunately, there is very little information on when and how signalling costs depend on condition; presently, this is the most important open question in the field of parent-offspring communication that requires empirical investigation.

## Supporting information

S1 Text Appendix. (DOCX)

S1 Notebook Interactive figure as *Mathematica* notebook. (NB)

S1 Video Video of interactive figure. (MP4)
