# Supplementary material for: One problem, too many solutions: How costly is honest signalling of need?
Source: PLoS One. 2019 Jan 11;14(1):e0208443. doi: 10.1371/journal.pone.0208443 (PMC6329501; doi:10.1371/journal.pone.0208443)
Supplement: S1 Text — (DOCX) [file pone.0208443.s001.docx]

**Appendix**

Supplementary Material

One problem, too many solutions: How costly is honest signalling of need?

Szabolcs Számadó^1,2^, Dániel Czégel^2,3^ and István Zachar^2,4,5^

^1^ RECENS „Lendület” Research Group, MTA Centre for Social Science, Tóth Kálmán u. 4., 1097 Budapest, Hungary

^2^ Evolutionary Systems Research Group, MTA, Centre for Ecological Research, Hungarian Academy of Sciences, Klebelsberg Kunó str. 3., 8237 Tihany, Hungary

^3^ Department of Plant Systematics, Ecology and Theoretical Biology, Eötvös Loránd University, Pázmány Péter sétány 1/c, 1117 Budapest, Hungary

^4^ MTA-ELTE Theoretical Biology and Evolutionary Ecology Research Group, Eötvös Loránd University, Department of Plant Taxonomy and Ecology, Pázmány Péter sétány 1/c, 1117 Budapest, Hungary

^5^ Center for the Conceptual Foundations of Science, Parmenides Foundation, Kirchplatz 1, 82049 Pullach/Munich, Germany

e-mail: [szamszab@ludens.elte.hu](mailto:szamszab@ludens.elte.hu)

# Different cost functions

There are two kinds of cost functions: (i) $f\left( x \right)$ specifies signal cost as the function of signal intensity (i.e. as a function of the offspring strategy); (ii) $L\left( c,z \right)$ specifies the cost as function of the quality of the offspring ($c$) and the parental investment ($z$). Note that $x\left( c \right)$ and $z\left( x \right)$ are the offspring and parental strategies respectively. At the honest signalling equilibrium there is a pair of optimal parent and offspring strategies ($z^{*}\left( x \right)$, $x^{*}\left( c \right)$) from which it does not worth departing unilaterally for any of the participants. Let’s denote $f\left( x^{*}\left( c \right) \right)=\tilde{f}\left( c \right)$ and $L\left( c,z^{*}(x^{*}\left( c \right)) \right)=L\left( c,\tilde{z}\left( c \right) \right)=\tilde{L}\left( c \right).$ While $f\left( x \right)$ is not known beforehand, one can calculate $L\left( c,z \right)$ at the equilibrium (see Section 2 in this Appendix), which also yields the value of $f\left( x \right)$ at the equilibrium, thus at equilibrium (where parties play their optimal strategies): $\tilde{L}\left( c \right)=\tilde{f}\left( c \right)$.

# Existence and stability of the signalling equilibria

The very same argument that was used by Nöldeke and Samuelson [1] can be used here to arrive at the second equilibrium signal cost. Only those parts should be checked where the explicit form of the cost function were used. Thus, concerning the existence of the proposition it remains to see whether the optimality condition can be derived with the new cost function (Eq. 14 of the main text).

$h\left( c,z^{*}\left( x^{*}\left( c \right) \right) \right)+\psi g\left( Z-z^{*}\left( x^{*}\left( c \right) \right) \right)-f\left( x^{*}\left( c \right) \right)=$ (A.1)

$$h\left( c,\tilde{z}\left( c \right) \right)+\psi g\left( Z-\tilde{z}\left( c \right) \right)-mL\left( \tilde{z}\left( c \right) \right)=$$

$$\psi\left( g\left( Z-\tilde{z}\left( c \right) \right)+\gamma h\left( c,\tilde{z}\left( c \right) \right) \right)-mh\left( c^{0},z^{0} \right)\geq$$

$$\psi\left( g\left( Z-z^{*}\left( c \right) \right)+\gamma h\left( c,z^{*}\left( c \right) \right) \right)-mh\left( c^{0},z^{0} \right)=$$

$$h\left( c,z^{*}\left( c \right) \right)+\psi g\left( Z-z^{*}\left( c \right) \right)-mL\left( z^{*}\left( c \right) \right)\geq$$

$$h\left( c,z^{*}\left( x \right) \right)+\psi g\left( Z-z^{*}\left( x \right) \right)-f\left( x \right).$$

The only change in this sequence is that Eq. 14 was used instead of Eq. 10 (both of the main text). One can see that at the second step after the rearrangement we obtain the parent’s maximisation problem as it was obtained by Nöldeke and Samuelson [1], using Eq. 10. Thus, Eq. 14 works, and the same argument can be applied.

The equilibrium condition for the offspring’s inclusive fitness is:

$h\left( c,\tilde{z}\left( c \right) \right)+\psi g\left( Z-\tilde{z}\left( c \right) \right)-{f(x}^{*}\left( c \right))\geq h\left( c,z^{*}\left( x\left( c \right) \right) \right)+\psi g\left( Z-z^{*}\left( x\left( c \right) \right) \right)-f(x\left( c \right)).$ (A.2)

Thus, we are looking for the signalling strategy $x^{*}\left( c \right)$ that optimizes the offspring’s inclusive fitness $v$, as a function of signal intensity $x$:

$v\left( x\left( c \right) \right)=h\left( c,z^{*}\left( x\left( c \right) \right) \right)+\psi g\left( Z-z^{*}\left( x\left( c \right) \right) \right)-f(x\left( c \right))$ (A.3)

Since $v$ is a functional of the function $x\left( c \right)$, the optimal $x$ can be found by using calculus of variations [2]: at the optimal $x\left( c \right)$, the variation of $v$ with respect to $x$ has to vanish, yielding the corresponding Euler-Lagrange equation:

$v_{x}=\left( h_{z}\left( c,\tilde{z}\left( c \right) \right)-\psi g_{y}\left( Z-\tilde{z}\left( c \right) \right) \right)\tilde{z}_{x}\left( c \right)-f_{x}\left( x^{*}(c) \right)=0$ (A.4)

After rearranging the solution function is:

$\left( h_{z}\left( c,\tilde{z}\left( c \right) \right)-\psi g_{y}\left( Z-\tilde{z}\left( c \right) \right) \right)\tilde{z}_{x}\left( c \right)=f_{x}\left( x^{*}\left( c \right) \right)$ (A.5)

We note, that in the paper of Nöldeke and Samuelson [1], there are consistent typesetting errors in A.2 and A.5 (and in between), where, presumably, some occurrences of $x\left( c \right)$ were replaced with $c$. We have remedied these errors in our Eqs. (A.2‑5).

The optimality condition for the parent as a function of resource allocation:

$\gamma h_{z}\left( c,\tilde{z}\left( c \right) \right)-g_{y}\left( Z-\tilde{z}\left( c \right) \right)=0$. (A.6)

This can be rearranged in two different ways:

$h_{z}\left( c,\tilde{z}\left( c \right) \right)=\frac{1}{\gamma}g_{y}\left( Z-\tilde{z}\left( c \right) \right)$, (A.6a)

$\gamma h_{z}\left( c,\tilde{z}\left( c \right) \right)=g_{y}\left( Z-\tilde{z}\left( c \right) \right).$ (A.6b)

As a result, equations A.5 and A.6 can be combined in two different ways. One can either substitute the right-hand side of A.6a or the left-hand side of A.6b into A.5. The first substitution gives the following equation:

$f_{x}\left( x^{*}\left( c \right) \right)=mg_{y}\left( Z-\tilde{z}\left( c \right) \right)\tilde{z}_{x}\left( c \right)$, (A.7)

where:

$f_{x}\left( x^{*}\left( c \right) \right)=mg_{y}\left( Z-z^{*}\left( x^{*}\left( c \right) \right) \right)\tilde{z}_{x}\left( c \right)$,

where $m=\left( 1/\gamma\right)-$. Integrating A.5 gives the cost function:

$f\left( x^{*}\left( c \right) \right)=k-mg\left( Z-\tilde{z}\left( c \right) \right)$ (A.8)

Since the least needy offspring should elicit the smallest resource transfer, that is, it should not engage in costly signalling, thus $k=mg(Z-z^{0})$:

$f\left( x^{*}\left( c \right) \right)=mg\left( Z-z^{0} \right)-mg\left( Z-\tilde{z}\left( c \right) \right)$, (A.9)

which is formally equivalent identical to Eq. 11.

The second substitution (i.e. substituting the left-hand side of A.6b into A.5) gives:

$f_{x}\left( x^{*}\left( c \right) \right)=mh_{z}\left( c,\tilde{z}\left( c \right) \right)\tilde{z}_{x}\left( c \right)$, (A.10)

where:

$f_{x}\left( x^{*}\left( c \right) \right)=mh_{z}\left( c,z^{*}\left( x^{*}\left( c \right) \right) \right)\tilde{z}_{x}\left( c \right)$,

where $m=1-$. Integrating gives:

$f\left( x^{*}\left( c \right) \right)=mh\left( c,\tilde{z}\left( c \right) \right)+k$. (A.11)

Again, we should scale this cost function in a way that the least needy young should have zero cost, thus $k=-mh\left( c,z^{0} \right)$. Substituting $k$ into A.6 gives:

$f\left( x^{*}\left( c \right) \right)=mh\left( c,\tilde{z}\left( c \right) \right)-mh\left( c,z^{0} \right)$, (A.12)

which is formally equivalent to Eq. 15. QED

# References

[1] Nöldeke, G. & Samuelson, L. 1999 How costly is the honest signaling of need? *Journal of Theoretical Biology* **197**, 527-539.

[2] Fox, C. 1950 *An introduction to the calculus of variations*, Courier Corporation.
